# Sickle Cell Trait: Is There an Increased VTE Risk in Pregnancy and the Postpartum?

**DOI:** 10.1371/journal.pone.0064141

**Published:** 2013-05-22

**Authors:** Sofya Pintova, Hillel W. Cohen, Henny H. Billett

**Affiliations:** 1 Department of Medicine, Mountt Sinai Medical Center, New York, New York, United States of America; 2 Department of Epidemiology and Population Health, Albert Einstein College of Medicine, Bronx, New York, United States of America; 3 Department of Medicine, Montefiore Medical Center, Bronx, New York, United States of America; Wayne State University School of Medicine, United States of America

## Abstract

Blacks are purported to have a higher venous thromboembolism (VTE) risk than whites. We hypothesized that this might be due, in part, to the greater presence of sickle cell trait (SCT) among blacks. We investigated whether the presence of SCT resulted in a higher VTE incidence in a population predisposed to VTE, the pregnant/postpartum women. Methods: Using a mirrored clinical database that prospectively gathered in- and out-patient information for the years 1998–2008, we collected demographic data, including hemoglobin electrophoreses, on all pregnant/postpartum non-Hispanic women who delivered at a large, diverse, urban hospital. We identified those women who developed VTE either while pregnant or postpartum during those 11 years. Charts initially identified as potential VTE cases were subjected to review to ensure accuracy of VTE coding. Results: Of 12,429 women, 679 non-Hispanic SCT black women, 5,465 non-Hispanic Hemoglobin AA (women with HbA as the only hemoglobin present on electrophoresis, with normal amounts of the minor hemoglobins) black women and 1,162 non-Hispanic HbAA white women were included in the analysis. SCT prevalence was high (11.1%) within this black population as compared to 8.3% in the general non-white population. Proportions with VTE were similar for black SCT and black HbAA groups: 0.44% for the SCT group, 0.49% for non-Hispanic black HbAA women. Black HbAA women had a non-significantly higher proportion of VTE than white HbAA women 0.49% vs 0.26% (RR 1.9, 95%CI:0.6,6.3, p = 0.28). Women with VTE were older than those without VTE (32.2 vs. 27.6 years, p = 0.0002) and the majority of VTE occurred postpartum in all groups, and significantly in the HbAA groups. There was no increase in the incidence of pulmonary emboli in the SCT group. Conclusion: In the largest analysis to date, we could not detect a meaningful difference in peripartum VTE incidence between women with and without sickle cell trait.

## Introduction

Venous thromboembolism (VTE) is a major cause of mortality in the United States. The genetic risk factors for VTE that have been identified, such as non-O blood groups, Factor V Leiden and Prothrombin 20210, are more common in whites than blacks [1]–[5]. This would favor the hypothesis that the burden of thromboembolic disease would be higher in white than in black individuals. However, there are emerging data that the VTE risk is not less and may indeed be higher in blacks, especially in black women during pregnancy and postpartum, as compared to whites [6]–[12]. A study investigating patients with diagnosed VTE reported a family history of VTE in blacks to be of a similar frequency as in whites (∼30%), suggesting a possibility of yet undiscovered genetic and/or acquired component(s) predisposing blacks to thrombotic events [6].

Sickle cell anemia is a genetic disease more prevalent in the black population and results in both an anemia and vaso-occlusion of blood vessels, typically post capillary venules. Sickle cell anemia has been associated with an increased incidence of VTE [13]. Whereas sickle cell anemia has been associated with a prothrombotic state, there are limited data to support a prothrombotic state in the heterozygote state, sickle cell trait (SCT) [14], [15]. Given that the prevalence of sickle cell trait (SCT) in the black community is high (6.5%–8.0%), even higher than the prevalence of factor V Leiden mutation among whites (5%), it is tempting to hypothesize that, if it exists, the prothrombotic proclivity of SCT may play a role in the higher incidence of VTE in the black population [13]–[19].

Another well-known prothrombotic state is pregnancy and the postpartum period, with rates of VTE estimated to be 4–10 fold that of the general population [20]–[25]. To date, no study has evaluated the potential role of SCT as a causative or contributing factor to venous thrombotic events in pregnancy. In order to investigate this aspect, we studied black and white non-Hispanic women with SCT and HbAA to determine whether women with SCT had a higher occurrence of thromboembolic events during pregnancy and the postpartum when compared to non-SCT women.

## Methods

### Study Population

Data were collected from Clinical Looking Glass (CLG). CLG is an interactive software application developed at Montefiore Medical Center that integrates demographic, clinical and administrative datasets and allows them to be reproduced in a programmable format for statistical access. Using International Classification of Diseases, 9^th^ revision, Clinical Modification (ICD-9-CM) codes, data on a pregnant woman’s first delivery within the designated eleven years between 1998 and 2008 were collected. Delivery date was considered the index date; the pregnancy/postpartum state was considered to be the time included by subtracting 42 weeks from the index date until eight weeks after the index date. Medical Center protocol regulations ensure that all pregnant women followed at the Medical Center have blood typing and hemoglobin (Hb) electrophoresis performed using high performance liquid chromatography (HPLC) with determination of %Hemoglobin A (HbA), %Hemoglobin A_2_ (HbA_2_ ), %fetal hemoglobin (HbF) and Hemoglobin S (HbS). Women with SCT were identified by hemoglobin electrophoresis results of %HbA> %HbS and by %HbS electrophoresis in the range of 30 to 45%. Women with a history of previously having had a %HbS >45% were not included, ensuring exclusion of participants with sickle cell disease who might be post-transfusion. HbAA (electrophoretic studies demonstrating HbA as the only hemoglobin present on electrophoresis, with normal amounts of the minor hemoglobins) was defined by %HbA electrophoresis values of ≥95.5%, thereby excluding women with β-thalassemia trait and an elevated %HbA_2_>3.5% with a %HbF of 1%.

Racial and ethnic demographics were obtained by self-identification, in accordance with NIH policy [26]. Subjects are provided with the option to select more than one racial category. Patients who selected “not available” option for race or ethnic identification were excluded. Patients self-reporting Hispanic or multiracial ethnicity were excluded since epidemiologic data suggest that Asians and Hispanics may have a lower occurrence of VTE and we were concerned that any increased effect of black race might be diluted by a decreased Hispanic risk [6]–[9]. In each group, patients with VTE were identified using ICD-9 diagnosis codes constructed with our Emerging Health Information Technology Team: 325, 415.1, 415.11, 415.19, 451, 451.0, 451.1, 451.11, 451.19, 451.2, 451.8, 451.81, 451.82, 451.83, 451.84, 451.89, 451.9, 452, 452.1, 453, 453.0, 453.2, 453.3, 453.40, 453.41, 453.42, 453.8, 453.9, 573.8. 671.2, 671.20, 671.21, 671.22, 671.23, 671.24, 671.3, 671.30, 671.31, 671.32, 671.33, 671.34, 671.4, 671.40, 671.41, 671.42, 671.43, 671.44, 671.5, 671.50, 671.51, 671.52, 671.53, 671.54, 673, 673.22, 673.23, 673.24, 673.8, 673.80, 673.81, 673.82, 673.83, 63.84. Chart reviews, both electronic and manual (as charts were available only as paper charts prior to 2005) were performed on all patients with a VTE diagnosis to ensure accuracy of the event and of the correct time frame. A thrombotic event had to correspond to an appropriate positive imaging modality (Venous Doppler ultrasonography, CT, MRI, V/Q scan) and the imaging study was cross-referenced to demonstrate an acute event within the set time frame. Patients with a past history of VTE who were thromboprophylaxed with unfractionated or low molecular weight heparin during pregnancy or the postpartum were excluded. Patients with suspected diagnosis of VTE without confirmatory imaging studies were also excluded. Patients who were found to have incorrectly coded conditions, such as venous stasis or stenosis, without evidence of current thrombosis were excluded. Patients temporarily anticoagulated while waiting for a VTE study which was subsequently read as negative were also excluded.

### Data Management and Data Analysis

We utilized the Biostatistical Consultative and Services Support Resource of the institutition’s Clinical and Translational Science Award (CTSA), which provides statistical, epidemiologic, and ethics support for the design of clinical and translational studies and collaborates with investigators on study conduct, analysis, interpretation and reporting of results. Results obtained from CLG were transferred to Excel spreadsheets (Microsoft Corp., Redmond, WA). SPSS (SPSS Inc., Chicago, IL) and Stata (Statacorp LP, College Station, TX) were also used for data entry and analyses. Two tailed t-tests assuming unequal variance were used to test differences in mean age after confirming that normality assumptions were not meaningfully violated. Chi square tests were used to test differences in proportions with VTE among those with and without SCT and rate ratios were reported as the ratio of proportions of SCT to HbAA. Sensitivity analyses were performed using the “worst case” assumption that all the unavailable charts were positive for DVT.

### Ethics Statement

This study was approved by Montefiore Medical Center Institutional Review Board (Approval #09-08-258E).

## Results

**Figure 1:**
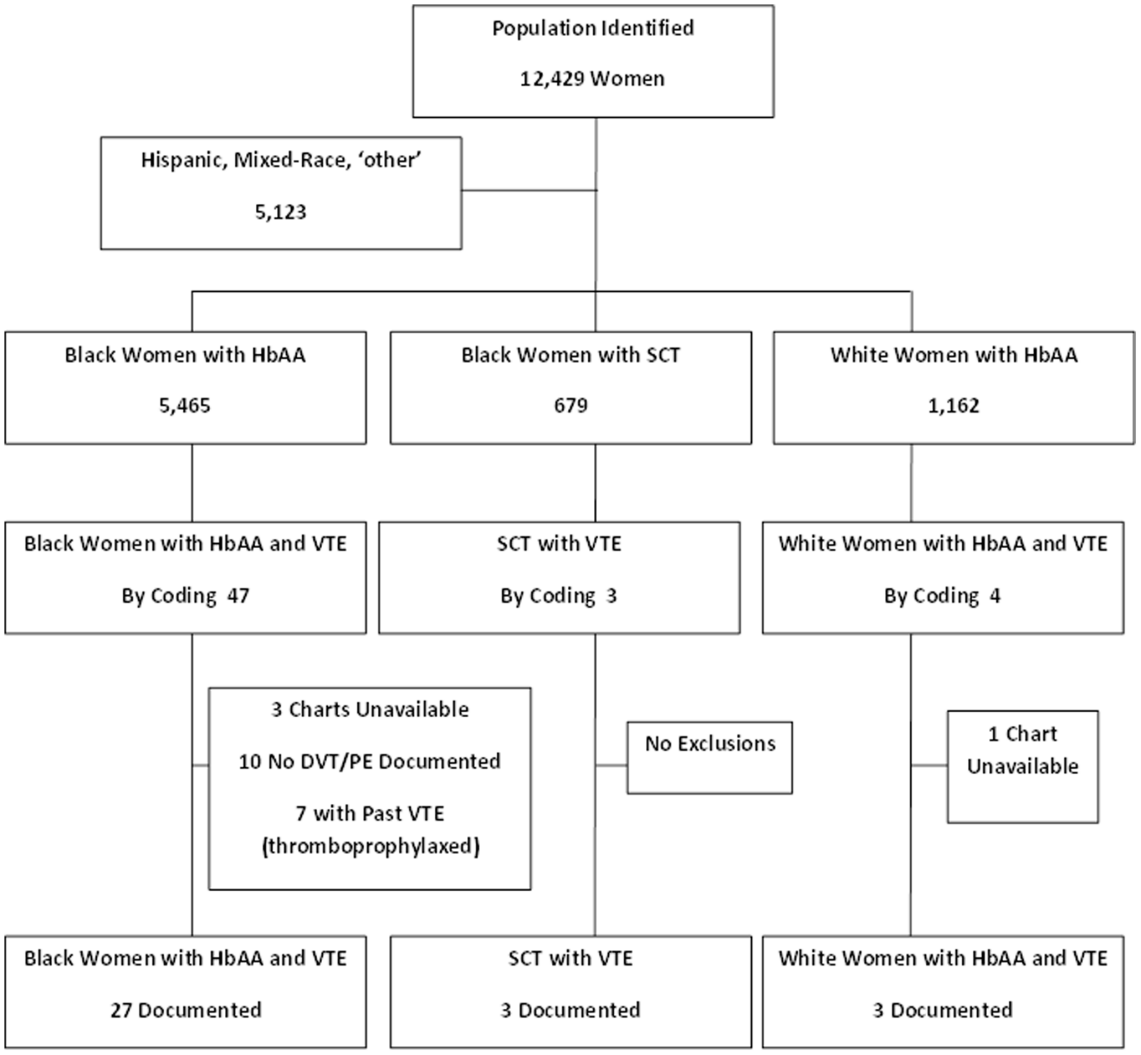
Flowchart of Study Patients.

Our database identified 12,429 women who delivered at Montefiore Medical Center, Einstein Campus, Bronx, NY between 1998 and 2008 (Table 1 and Figure 1) and who had a hemoglobin electrophoresis performed. The prevalence of SCT in this population was 8.3%. In this original cohort, 5,123 individuals classified themselves as Hispanic, mixed-race, ‘other’ or were unclassified. These were excluded. Of the remaining 7,306 women, 1,162 were white women with HbAA. The remaining black cohort consisted of 6,144 women, of which 5,465 black women had HbAA and 679 black women had SCT. In this non-Hispanic, more homogeneous cohort, the prevalence of sickle cell trait in the black population was found to be 11.1%.

**Table 1:** Characteristics of the patient population.

	Non-Hispanic	Hispanic	Total
SCT			
Black	679	58	737
Multiracial	10	138	148
SCT Total	689	196	885
HbAA			
Black	5,465	524	5,989
Multiracial	375	3,397	3,772
White	1,162	621	1,783
HbAA Total	7,002	4,542	11,544
TOTAL	7,691	4,738	12,429

Seven women with a previous history of VTE were thromboprophylaxed during their pregnancy/postpartum period; these women were excluded from analysis. Of the 7,306 patients included in the analysis, only 33 women (0.45%) had a VTE during the designated time period. Initially this proportion appeared to be higher at 0.7% but when charts were reviewed for accuracy, 21 of these “VTE” diagnoses were excluded: 10 patients had no evidence of VTE despite positive diagnostic coding: 5 of these had no VTE documented in chart at all and 5 women who were suspected of having a DVT had subsequent negative imaging. Four charts were unavailable (3 black HbAA and one white HbAA). After chart review, only 3 black patients with SCT (0.44%), 3 white HbAA patients (0.26%) and 27 black patients with HbAA (0.49%) were found to have documented evidence of VTE (Figure 1).

**Figure 2:**
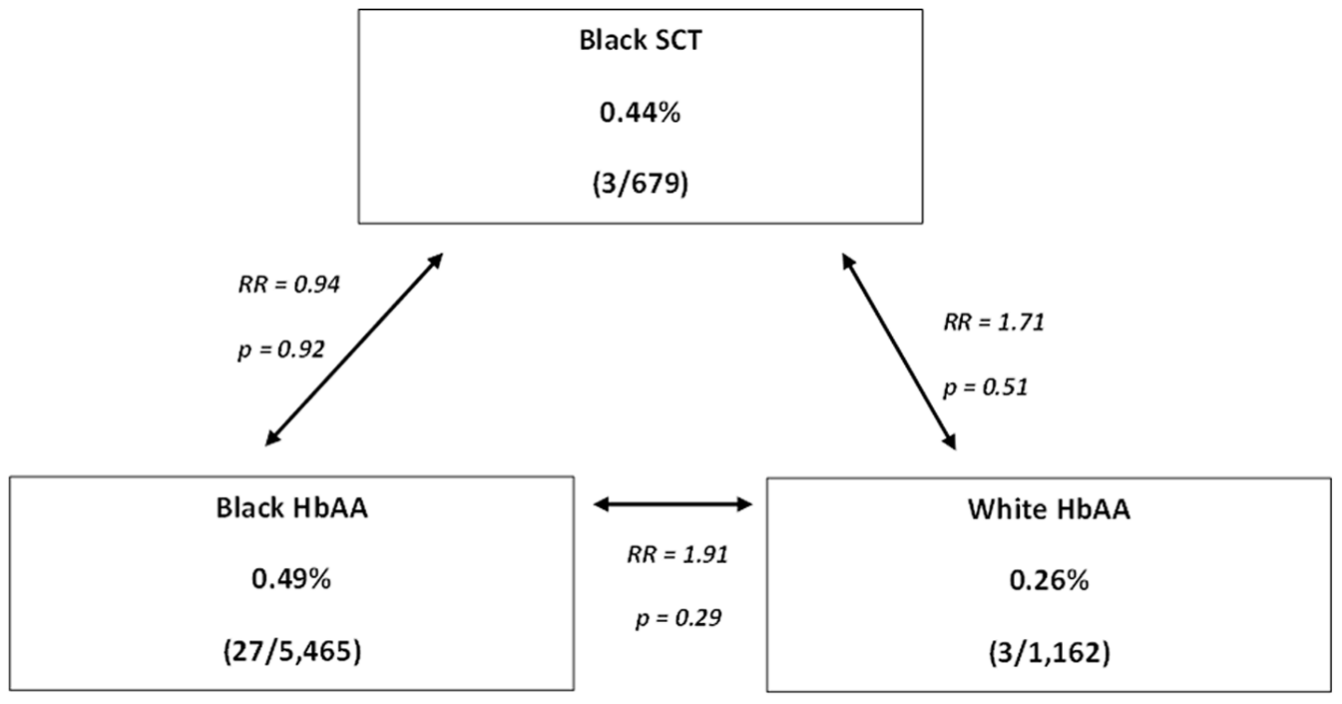
Rate Ratios (RR) of VTE in Black Sickle Cell Trait, Black HbAA and White HbAA.

Although there was a trend toward a higher incidence of VTE in blacks, this increase was not statistically significant (RR 1.9, 95%CI: 0.6, 6.3, p = 0.28). As detailed in Figure 2, the proportion of VTE in pregnant/postpartum women with SCT was similar to that of HbAA pregnant/post-partum women (0.44% for SCT vs 0.49% for HbAA p = 0.92). When these data were reanalyzed to include the possibility that all the unavailable charts were positive for DVT, the incidence in HbAA women rose to 0.51% while the VTE incidence for SCT still remained unchanged at 0.44%. More importantly, if all the seven women who were thromboprophylaxed and excluded from the analysis were, instead, included and hypothesized to have had a VTE, the incidence rose only for black HbAA to 0.68% and did not change the 0.44% incidence of SCT.

The types of thromboses were varied for all groups. In the SCT group, 2 patients were found to have lower extremity VTE and 1 patient had a pelvic vein thrombosis. In the white HbAA group, the thrombotic events were 1 pulmonary embolus, 1 superficial thrombosis, and 1 lower extremity VTE. In blacks with HbAA, where 27 patients were found to have VTEs, 10 had lower extremity VTE, 8 had pulmonary emboli, 2 had both lower extremity VTE and pulmonary emboli, 2 had septic thrombophlebitis, 1 had renal vein thrombosis, 1 had dural sinus thrombosis, 2 experienced only superficial vein thrombosis, and 1 patient had an upper extremity VTE and pulmonary emboli. We saw no increase in pulmonary embolism in SCT patients.

Age difference between pregnant black HbAA and pregnant white HbAA was significant (27.2 vs. 29.0 yrs., p<0.000001). Importantly, the difference in ages between patients with and without VTE for the whole cohort (32.2 yrs with VTE vs. 27.6 yrs without VTE, p = 0.002) and for the black population (32.4 yrs. vs. 27.2 yrs. respectively, p = 0.003) were also significant. Although these age differences held for the white and the SCT cohorts, the VTE rates for these women (3 per set) were too low for assessment of significance (Table 2).

**Table 2:** VTE: Ages of cohorts with and without VTE.

	No VTE	VTE	p value
White HbAA (yrs +SD)	29.0±5.9*	**30.3±2.9**	**0.52**
Black HbAA (yrs +SD)	27.2±6.5*	**32.4±8.3**	**0.003**
Black SCT (yrs +SD)	**27.9±6.5**	**32.3±9.0**	**0.48**
Total (yrs +SD)	**27.6±6.4**	**32.2±7.8**	**0.002**

Thrombotic events occurred disproportionately in the post-partum period; 61% of VTE occurred within 8 weeks of delivery. Postpartum VTE accounted for 67% of the total VTE for SCT, 100% of total white HbAA VTE and 55.5% of total black HbAA thrombotic events.

Although the incidence of VTE was quite low, assuming that the true population proportion of VTE among HbAA women was.0045% we had 92% power to detect a four-fold increase for SCT women, 79% power to detect a three-fold increase.

## Discussion

Several studies have described a higher rate of VTE in blacks and some have investigated the association of SCT with thromboembolic disease [6]–[11], [16]–[18]. In support of the hypercoagulability hypothesis, Westerman noted increased levels of prothrombotic markers such as D-dimers, TAT (thrombin-antithrombin complexes) and the prothrombin cleavage fragment F1.2 in patients with SCT compared to HbAA controls [27]. Other investigations have attempted to link clinical incidence of VTE to SCT, suggesting that sickle cell trait may predispose to a higher occurrence of thrombotic events [16]–[18]. Early data from Heller at al. in the 1970’s demonstrated an increased incidence of pulmonary embolism in black men with SCT compared to controls (2.2% vs. 1.5%, RR 1.46, 95%CI:1.14, 1.89, p<0.001). However, these data largely depended on the clinical diagnosis of PE before more precise imaging studies, such as spiral CT, became widely available [16]. A more recent study by Austin et al. examined the association of SCT among inpatient blacks with venous thromboembolism in the Atlanta, Georgia area but compared them to outpatient controls. The larger group, that with DVT, showed no increased risk but subjects with pulmonary embolism were three times as likely to have SCT compared to controls [18]. In our study, with the same incidence of hospitalizations, we found no such increase in pulmonary emboli. Another publication examining a subset specifically looked at VTE risk in black women with SCT. Women with SCT using hormonal contraception had a slightly higher rate of VTE compared to women without SCT but the difference was not statistically significant [17].

We analyzed the data first and foremost for SCT vs. HbAA. We were able to identify a large cohort of pregnant women with SCT and a very large cohort of black pregnant women with HbAA. We did not find an increased occurrence of VTE in women with SCT during pregnancy/postpartum when compared to pregnant/postpartum black and/or white women with HbAA. If there truly is a black:white difference in VTE rate, our data do not support the hypothesis that such a difference is due to a prothrombotic effect of SCT or that SCT is, in and of itself, prothrombotic. We did not examine for other specific risk factors and it is certainly possible that other VTE risk factors could alter results. We cannot exclude the fact that this may have contributed to the difference between white and black HbAA or between black HbAA and black SCT.

As is customary for studies involving race and ethnicity, race was self-declared. There is evidence that self-reporting is accurate for this purpose [28]. We attempted to define a black population that excluded, to the best of our ability, multiracial and Hispanic groups. This may have contributed to the increased prevalence of SCT in our black cohort, at 11.1%, as compared to the 8.3% that would have been the prevalence had we included the black multiracial and Hispanic patients. The issue of what constitutes the “black” population may play a role in future studies that attempt to define factors influencing thrombotic risks in specific groups.

This study is a “retrospective-prospective study:” Data were collected by the Montefiore Medical Center Clinical Information System prospectively, but our study question and analyses were carried out retrospectively. This allowed an unbiased prospective assessment of the proportion of incident VTEs rather than attempting to start with patients already diagnosed with VTE [17], [18]. By starting out with the delivery date as index date, we could not take into consideration women with multiple miscarriages as evidence for thrombosis in SCT. However, were this a significant issue, this should have resulted in an increased age at delivery for SCT patients – something we did not find. Our analysis affirms the positive correlation of age with VTE irrespective of SCT status. Although previous reports have suggested age as a significant risk factor for pregnant women with VTE after the age of 35 yrs [19], our analysis demonstrates that, even within the “younger” set, the older woman has a significantly increased VTE risk in pregnancy/postpartum.

There are some limitations for these data analyses. The primary limitation derives from the low VTE rate. Our cohort included only pregnant/postpartum women who had had a hemoglobin electrophoresis, but our study population was young (range 27–32 years), making the general incidence of VTE low. Although ours is the largest study of its kind to date, power to detect small differences was still an issue due to the low VTE rate. Indeed, although the white population has a VTE rate that is similar to that previously reported for our general obstetrical population in the Medical Center, the black cohort with its higher VTE incidence was still too low to allow for sufficient power [29]. Although similar to what has been previously published for this population [20], these SCT patients may be at a significantly lower risk than other published study SCT populations in other situations and with older age ranges [17]–[18], [30].

However, assuming that the true population proportion of VTE among HbAA women is 0.45%, we had 92% power to detect a four-fold increase in VTE risk for SCT women and we had 79% power to detect a three-fold increase. We had <40% power to detect increased risks which were less than 2-fold. The clinical significance of a less-than-two-fold increase in risk for an event which has a low absolute risk is difficult to assess and we suggest that this increase might not be enough to advocate changes in thromboprophylaxis regimens but that more investigation into this therapeutic aspect is warranted.

It is also possible that other risk factors for VTE including BMI and C-section procedures were not balanced between the comparison groups. While we have no reason to believe such imbalances would impact the results, we did not have the data available to definitively rule out the possibility of confounding.

The results of our study suggest that the role of SCT as an inherited prothrombotic mutation remains unclear, especially during pregnancy and postpartum periods. Additional large studies as well as meta analyses will be needed to better characterize the true impact of SCT on venous thromboembolism in pregnancy/postpartum.
